# Microstructure and Mechanical Properties of Gradient Interfaces in Wire Arc Additive Remanufacturing of Hot Forging Die Steel

**DOI:** 10.3390/ma16072639

**Published:** 2023-03-27

**Authors:** Mao Ni, Zeqi Hu, Xunpeng Qin, Xiaochen Xiong, Feilong Ji

**Affiliations:** 1 Hubei Key Laboratory of Advanced Technology for Automotive Components, Wuhan University of Technology, 122 Luoshi Road, Wuhan 430070, China; 2 School of Automotive Engineering, Wuhan University of Technology, Wuhan 430070, China; 3 Hubei Collaborative Innovation Center for Automotive Components Technology, Wuhan 430070, China; 4 Hubei Longzhong Laboratory, Wuhan University of Technology (Xiangyang Demonstration Zone), Xiangyang 441000, China

**Keywords:** microstructure, gradient interface, hot forging die remanufacturing, wire arc additive manufacturing

## Abstract

Hot forging dies are subjected to periodic thermal stress and often fail in the forms of thermal fatigue, wear, plastic deformation, and fracture. A gradient multi-material wire arc additive remanufacturing method for hot forging dies was proposed to extend the service life of hot forging dies and reduce total production costs. The properties of multi-material gradient interfaces play a critical role in determining the overall performance of the final products. In this study, the remanufacturing zone of a hot forging die was divided into three deposition layers: the transition layer, the intermediate layer, and the strengthening layer. Experiments of wire arc additive manufacturing with gradient material were conducted on a 5CrNiMo hot forging die steel. The microstructure, microhardness, bonding strength, and impact property of gradient interfaces were characterized and analyzed. The results revealed that the gradient additive layers and their interfaces were defect-free and that the gradient interfaces had obtained a high-strength metallurgical bonding. The microstructure of the gradient additive layers presented a gradient transformation process of bainite-to-martensite from the bottom to the top layer. The microhardness gradually increased from the substrate layer to the surface-strengthening layer, forming a three-level gradient in the range of 100 HV. The impact toughness values of the three interfaces were 46.15 J/cm^2^, 54.96 J/cm^2^, and 22.53 J/cm^2^, and the impact fracture morphology ranged from ductile fracture to quasi-cleavage fracture. The mechanical properties of the gradient interfaces showed a gradient increase in hardness and strength, and a gradient decrease in toughness. The practical application of hot forging die remanufactured by the proposed method had an increase of 37.5% in average lifespan, which provided scientific support for the engineering application of the gradient multi-material wire arc additive remanufacturing of hot forging dies.

## 1. Introduction

Hot forging is a reliable and relatively economical forming process for producing metal parts in the automobile and aerospace industries [1,2], especially high-performance, lightweight structural components such as aluminum alloys [3,4,5] and titanium alloys [6,7]. During the working process, hot forging dies are subjected to periodic thermal stress due to the repeated extrusion and impact of high-temperature metal [8]. The surface of dies often fails in the forms of thermal fatigue, wear, plastic deformation, and fracture, which seriously affect the service life of dies [9,10]. In addition, the production of a new die is costly in terms of materials and time. In order to increase the service life of hot forging dies and save on production costs, it is usually necessary to repair and remanufacture them for many times within their life cycle [11]. The thickness of the failure cavity of a die can be up to 10–20 mm, and traditional surface modification technologies such as brush plating and thermal spraying are not suitable for remanufacturing large hot forging dies due to their lower deposition thickness [12].

The recent rapid development of directed energy deposition (DED) additive manufacturing technology provides a novel conception for remanufacturing large metal components [13,14]. The DED process is categorized into powder-feed and wire-feed types according to the feedstock [15]. The powder-based DED processes generally use a laser as the heat source [16,17], while the wire-based DED processes utilize an electric arc, a plasma arc, a laser, or an electron beam as the heat source [13]. Among these DED processes, wire arc additive manufacturing (WAAM), which associates electric arc as the heat source and welding wire as the feeding material, has distinctive features of low capital costs, high deposition efficiency (50–130 g/min), and material utilization [18,19]. It has been widely applied to the field of die repair and remanufacturing, especially the remanufacturing of hot forging dies [20,21,22]. According to the principle of stacking materials layer upon layer, the cavity of the failure die is regarded as the substrate; then, the gradient layers with desired properties and characteristics can be easily remanufactured through depositing the welding wires of multiple materials, which is a green and intelligent remanufacturing technology in the context of the circular economy [23].

The complex working conditions of hot forging dies require that the surface material of the cavity exhibits excellent high-temperature wear resistance and fatigue resistance properties, while the inner material needs to possess a certain degree of toughness and good strength [24]. For traditional homogenous material dies, it is challenging to simultaneously achieve high hardness and good toughness. Therefore, for the purpose of further extending the service life of a die, it is essential to partition the region to be remanufactured according to the temperature and stress fields of the die in the service process; then, different materials can be used for filling. The remanufactured hot forging die with outstanding comprehensive performance can be obtained by means of the optimization and matching of gradient materials. Aiming at solving this problem, Wang et al. [25] designed a long-service-life hot forging die with a multilayer metal structure. Through finite element simulation analysis, it is proved that using a multilayer metal structure can effectively reduce the peak temperature of the cavity surface and relieve the overall stress of the die. Lu et al. [20] conducted surface welding on casting steel for die fabrication. The simulations of the surface welding process and forging process recommended a welding thickness of 16 mm to synchronously improve the overall performance of forging dies and reduce the cost of die fabrication. Zhang and Shen et al. [26,27] proposed bimetal-layer surfacing technology to further elongate the service life of hot forging die. These studies have proven that the gradient surfacing remanufacturing method can extend the lifespan of dies and reduce the total cost. The use of robotic WAAM technology allows the efficient and controlled deposition of various materials to be achieved in specific regions of the die being repaired, making it an ideal method for remanufacturing hot forging dies with gradient materials [11,21].

Some scholars have investigated the microstructure and performance of materials of hot forging dies remanufactured using WAAM. Wang et al. [28] studied the microstructure, hardness, and tensile properties of die steel H13 fabricated by WAAM. Chen et al. [29] analyzed the relationship between the microstructure and wear resistance of surfacing layers with different flux-cored wires. Shen et al. [27] proposed a bimetal-layer weld surfacing method for hot forging dies and investigated the microstructural evolution and mechanical properties achieved with different surfacing alloy materials. However, few studies have focused on interfacial properties in multi-material additive manufacturing. For gradient materials via the WAAM process, Chandrasekaran et al. [30] adopted duplex stainless steel and carbon manganese steel to manufacture marine risers with gradient properties using CMT-based GMAW. They found that a sufficient diffusion of alloying elements occurred at the interface, leading to an increase in mechanical strength. Wang et al. [31] investigated the grain feature, phase change, and mechanical properties of TA15/TC11 gradient material along the additive direction. It was indicated that the composition segregation was induced at the interface. However, few studies are reported on the microstructure and properties of the multi-material gradient interfaces of hot forging die steel remanufactured using WAAM. The use of gradient multi-material remanufacturing for hot forging dies is a relatively new research field, and understanding the microstructure and properties of multi-material gradient interfaces using the WAAM process is crucial to determine the overall performance of the final parts. 5CrNiMo, with good hardness, toughness, hardenability, wear resistance, and tempering resistance, is the most widely used hot forging die steel [15,25]. In this paper, a gradient multi-material wire arc additive remanufacturing method for hot forging dies is proposed. The remanufacturing zone of a hot forging die is divided into three deposition layers based on temperature and stress analyses; then, three kinds of welding wires are deposited on the 5CrNiMo hot forging die steel using the WAAM process. The microstructure and properties of multi-material gradient interfaces are evaluated and discussed in detail. Finally, the practical remanufacturing application of a hot forging die is conducted to verify the feasibility of manufacturing and the overall performance of the gradient layers.

## 2. Experimental Procedure

### 2.1. Experimental Setup

**Figure 1:**
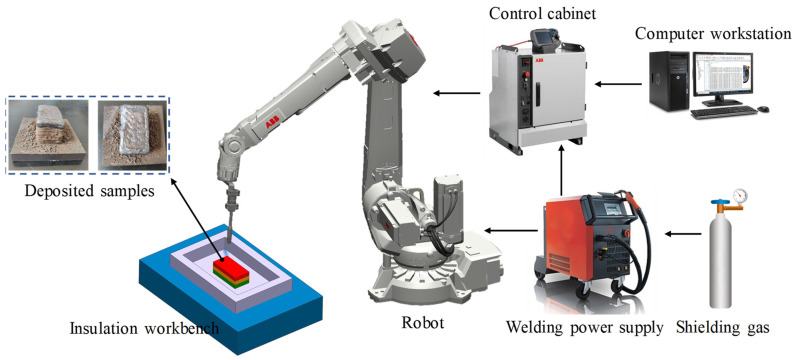
Schematic diagram of the robotic GMAW-based additive manufacturing system.

Figure 1 depicts the experimental system, which was composed of a welding power system, a computer workstation, a robot system, and an insulation workbench. A programmable digital LORCH S8 welding supply (LORCH, Baden-Württemberg, Germany) in GMAW mode was employed to control the welding process parameters. The motion path code of the robot was generated by the computer workstation, and a 6-axis ABB robot (IRB 1410) (ABB Robotics, Zurich, Switzerland) equipped with a welding torch was implemented to execute the designed additive trajectory. A control cabinet was used to coordinate the robot and the welding power supply. A mixture of shielding gas (18% CO_2_ and 82% Ar) was used to improve the arc and droplet transfer stability during the welding process [32].

### 2.2. Materials

**Figure 2:**
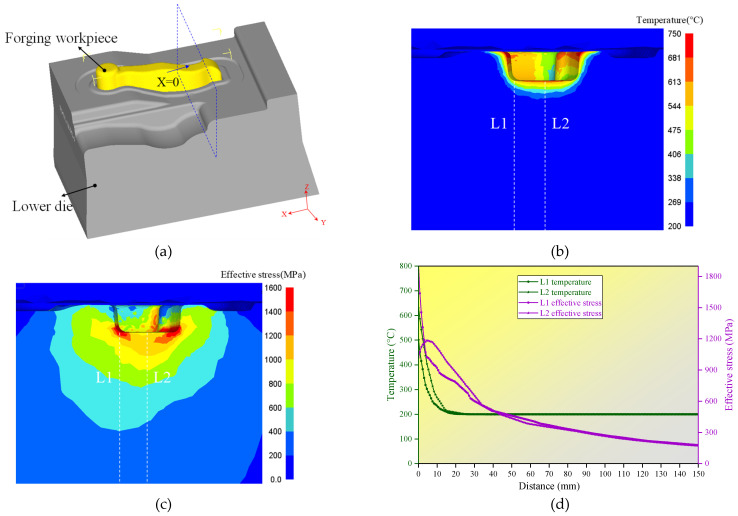
Three-dimensional model and temperature–stress fields of the die. (**a**) Three-dimensional model. (**b**) Temperature. (**c**) Effective stress. (**d**) Temperature and stress at L1 and L2.

To define the gradient structure of multi-material wire arc additive remanufacturing, a finite element model (FEM) of the hot forging forming process was developed using DEFORM-3D software (DEFORM-3D Ver 11.0, Scientific Forming Technologies Corporation, OH, USA) to analyze the temperature and stress distribution in the hot forging die. The workpiece and die materials were 42CrMo and 5CrNiMo, respectively. Figure 2a presents the 3D model of the workpiece and lower die. The simulation parameters are listed in Table 1 and Table 2.

**Figure 3:**
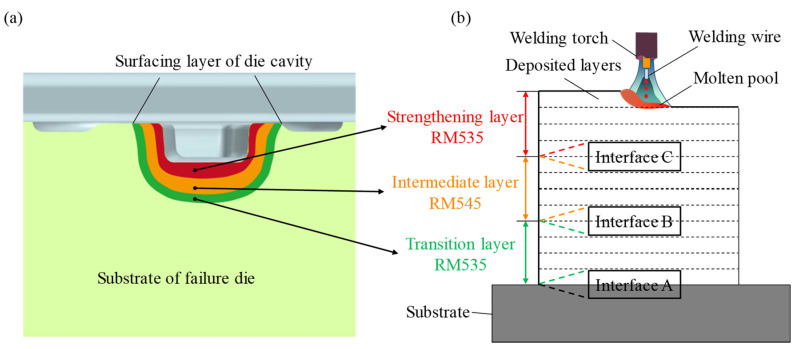
Schematic diagram of multi-material distribution in GAL: (**a**) structural division of the die; (**b**) experiment simplification of GMAW-based additive manufacturing and gradient interfaces of deposited layer.

The temperature and effective stress fields of the die after 11 continuous forging cycles are depicted in Figure 2b,c. It can be observed that the temperature and stress of the die were extremely unevenly distributed. The high-temperature regions were mainly concentrated on the upper edge of the cavity surface, while the high-stress regions were located near the interior of the lower edge of the cavity. In addition, the values of the temperature and stress of two reference lines L1 and L2 (marked in Figure 2b,c) are indicated in Figure 2d. It can be found that the relative gradient of the temperature was greater than that of the stress, and both temperature and stress tended to decrease with the increase in the distance from the cavity surface. Therefore, the additive welding layer of the remanufacturing zone could be divided into three gradient layers from the surface to the inside: the strengthening layer, intermediate layer, and transition layer. Herein, these three layers are collectively referred to as the gradient additive layer (GAL). The strengthening layer is in direct contact with the workpiece and cyclically subjected to high temperature and stress, as well as severe friction loads. The material used for this layer should have high thermal stability and excellent temperature wear resistance. The intermediate layer experiences high-temperature and stress, and the corresponding material should be simultaneously provided with good impact toughness and high temperature strength. The transition layer, where temperature and stress are relatively low and tend to gradually reach a balance, serves for the smooth transition of element composition and mechanical performance between the substrate metal and the repairing layer, as well as provides toughness to buffer the surface stress. The procedure for region division is as follows: First, the remanufacturing zone and substrate are divided based on the initial temperature of the die and the yield strength of the substrate material. Next, the strengthening layer is distinguished from the rest of the remanufacturing zone by considering the temperature fluctuation, custom high temperature, and yield strength of the intermediate layer material. Finally, the intermediate layer and transition layer are determined according to the custom medium temperature and the yield strength of the transition layer material. The designed structure of the gradient layers of the remanufacturing zone is illustrated in Figure 3a. Flux-cored welding wires of RM535, RM545, and RM650 (co-developed by the author’s team and an enterprise in China) with a diameter of 1.6 mm were selected as the materials for the transition layer, intermediate layer, and strengthening layer, respectively. The material chemical composition is listed in Table 3. When designing these three welding wires, the content of C and the strong carbide-forming elements were mainly considered, such as Cr, Mo, W, and V. Since most of the hot forging dies failed in the form of wear, greater attention was paid to the strength index. Meanwhile, we also hoped to improve the toughness while ensuring the strength, especially for the inside of the die. Ni is beneficial to the plasticity and toughness of Fe-based alloys and has little effect on their strength. Therefore, the Ni content in the transition layer was slightly higher. On the whole, the materials of the additive remanufacturing layers have the characteristics of low carbon and high alloy. In the experiment, the curved die cavity was simplified to a planar substrate of 5CrNiMo steel with a thickness of 15 mm, as shown in Figure 3b, and the deposited samples are shown in Figure 1. This paper focuses on the microstructure and properties of the gradient interfaces described in Figure 3b.

The 5CrNiMo substrate was subjected to preheating treatment at a temperature of 450 °C for 2 h prior to deposition, using an insulated worktable to maintain an interlayer temperature of at least 350 °C during the depositing process [25]. Upon completion of the deposition process, the samples were cooled to room temperature before being tempered at 550 °C for 3 h. The shielding gas was a gas mixture of 18% CO_2_ and 82% Ar at a flow rate of 16 L/min. The wire extension and the contact tip-to-workpiece distance were maintained as 12 mm and 15 mm, respectively, with a constant overlap ratio of 50% between adjacent beads. RM535, RM545, and RM650 were sequentially deposited from the bottom to the top, and the thickness of each deposition layer was 12–14 mm. Figure 3b illustrates the schematic diagram of the GMAW-based additive manufacturing process and the gradient interfaces of the deposited layer, with interface A, interface B, and interface C representing the fusion zones of substrate–transition layers, transition–intermediate layers, and intermediate–strengthening layers, respectively. The welding parameters are listed in Table 4.

### 2.3. Testing and Characterization

**Figure 4:**
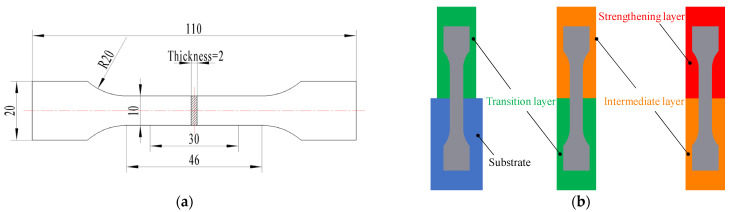
Size and sampling location of tensile test specimens. (**a**) Tensile specimen (unit: mm). (**b**) Sampling locations.

The specimens of the WAAM sample were cut using electric spark wire machining, with a cross-section size of 10 × 10 mm. The metallographic specimens were then etched with tri-acid ethanol solution (saturated trinitrophenol:nitric acid:hydrochloric acid:ethanol = 2:1:2:5) for a period ranging from 10 s to 30 s. The microstructure of the gradient interfaces was analyzed using optical microscopy. The microhardness distribution in the GAL was measured using an HXD-1000TM semi-automatic Vickers microhardness tester, with a load of 500 g and a dwell time of 10 s (Standard ID: GB/T 4340.1-2009). The hardness test was conducted at every 0.5 mm interval. The bonding strength of the gradient interfaces was tested through the tensile method, using specimens cut according to Chinese national standard GB/T 228.1-2010 (the specimen size is shown in Figure 4, with the interface between adjacent layers being located at the center of the specimen). Additional multi-layer additive experiments were conducted to obtain more tensile specimens, due to their length being too great. The tensile tests were carried out using an SHT4106 electro-hydraulic servo universal testing machine.

**Figure 5:**
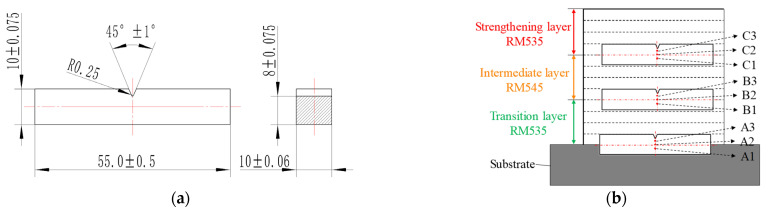
Sampling locations and observation points of impact fractures. (**a**) Impact specimen (unit: mm). (**b**) Sampling locations and fracture observation points.

To analyze the toughness and fracture characteristics of the gradient interface of the additive deposition layers, impact specimens with a size of 10 × 10 × 55 mm and a V-notch were sampled at the interface. The impact testing standard is Chinese national standard GB/T 229-2007. Each specimen was tested three times, and the average value was obtained. The fracture morphology was observed using a JSM-IT300 scanning electron microscope (SEM). The sampling positions and observation points of the impact fractures are shown in Figure 5, with observation points A1 and A3 of interface A being located near the substrate and transition layer, respectively, and point A2 being at the center of interface A. Similarly, the observation points on interfaces B and C were deduced in turn (marked in Figure 5b).

## 3. Results and Discussion

### 3.1. Microstructure of Gradient Interfaces

**Figure 6:**
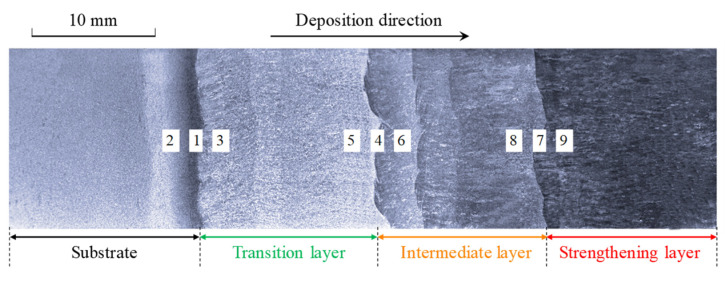
The macroscopic view of the GAL. Numbers 1–9 indicated the observation positions of microstructure in Figure 7, Figure 8 and Figure 9.

**Figure 7:**
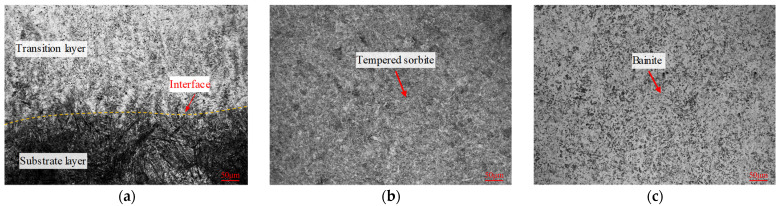
Microstructure of interface A. (**a**) Interface A (position 1). (**b**) Substrate layer (position 2). (**c**) Transition layer (position 3).

**Figure 8:**
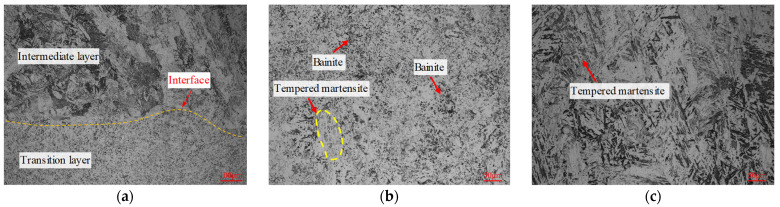
Microstructure of interface B. (**a**) Interface B (position 4). (**b**) Transition layer (position 5). (**c**) Intermediate layer (position 6).

**Figure 9:**
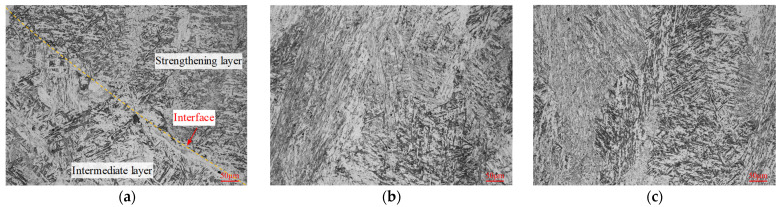
Microstructure of interface C. (**a**) Interface C (position 7). (**b**) Intermediate layer (position 8). (**c**) Strengthening layer (position 9).

The overall macroscopic view of the GAL, which was etched with 4% nitric acid ethanol solution, is shown in Figure 6 to evaluate the deposition quality and indicate the sampling position of the microstructure. Figure 7, Figure 8 and Figure 9 present the microstructure of interfaces A, B, and C after tempering, and each sampling position can refer to the number marks in Figure 6. It can be found that metallurgical bonding was achieved near the interfacial fusion zone of each adjacent additive layer. No defects such as cracks, slag inclusions, or voids were observed at both the macro-scale and micro-scale.

In Figure 7a, a clear difference in the microstructure of the two side materials near interface A can be seen. The microstructure of the transition layer at interface A was composed of bainite and fine acicular martensite. This was likely due to the preheating of the 5CrNiMo substrate before depositing and the influence of the molten pool temperature by the subsequent additive layer, which reduced the cooling rate of the entire transition layer and promoted the formation of bainite. On the side of the substrate layer, the testing surface was deeply corroded, and some coarse martensite appeared. This may have been due to the first layer of weld beads having partially remelted the substrate layer. The Cr and Mo in the transition layer tended to form stable carbides with C. As a result, the migration and diffusion of C near interface A were reduced. Figure 7b shows the microstructure of the substrate layer about 2 mm below interface A. The 5CrNiMo substrate was composed of fine, uniform tempered sorbite and a small amount of ferrite and carbide particles [33]. Figure 7c shows the microstructure of the transition layer about 2 mm above interface A. The transition layer was mainly composed of granular bainite and a few fine, tempered martensite structures. Bainite structures present good strength, high toughness, and excellent thermal-shock resistance, which can cushion the impact load from the die surface.

Figure 8a shows the microstructure near interface B. The microstructure of the adjacent materials has a distinct morphology, and the interface is clearly distinguishable. In the region below interface B (as shown in Figure 8b), the microstructure of the transition layer was mainly composed of granular bainite, tempered martensite, and dispersed carbide particles. Compared with the above results (Figure 7c), the content of tempered martensite was slightly higher, which may be due to the differences in the thermal process of the material at different additive heights. The higher the additive layer height, the faster the cooling rate, which is more conducive to martensite transformation. The microstructure of the intermediate layer side above interface B (as shown in Figure 8c) mainly comprised lath martensite, fine carbides, and a small amount of bainite. During the tempering process, the high content of strong carbide-forming elements in the intermediate layer leads to the precipitation of more carbides in the martensite, further enhancing the overall hardness and wear resistance.

The microstructure of interface C is presented in Figure 9. It can be noted that the difference in the morphology of the microstructure between the two side materials is small, and the interface is not easy to identify. This was due to the relatively similar type and content of the material elements in the intermediate and strengthening layers. Compared with the transition and intermediate layers, the strengthening layer exhibits elevated contents of the alloying elements C, Cr, and Mo. As a consequence, this elevation in alloying element content is responsible for a reduction in the temperature range of the bainite transformation, as well as a shift in the nose of the bainite transformation to longer times [34]. In other words, the tendency towards bainite transformation decreases while the critical cooling rate required for martensite transformation decreases. Additionally, the strengthening layer is situated at the surface of the additive layers and experiences a more rapid cooling rate, thereby favoring the martensite transformation process. The microstructure of interface C and the strengthening layer was composed of a mixture of acicular martensite and lath martensite, with a large amount of uniform carbide-strengthening phases formed by Mo and Cr between martensite structures.

### 3.2. Microhardness and Bonding Strength of Gradient Interfaces

**Figure 10:**
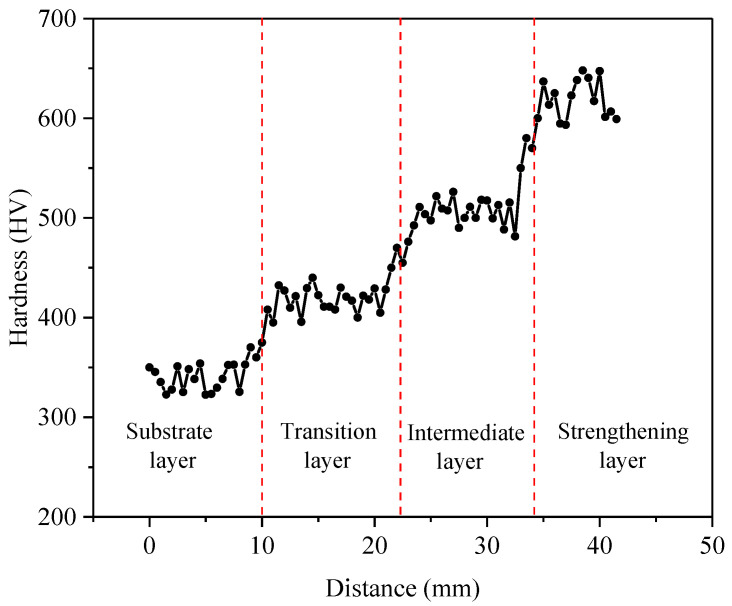
Microhardness distribution of the GAL.

Figure 10 shows the microhardness distribution of the GAL. It can be found that the microhardness gradually increased from the substrate layer to the surface strengthening layer. The average microhardness values of the substrate layer, transition layer, intermediate layer, and strengthening layer were 331.6 ± 13.4 HV, 414.7 ± 17.3 HV, 506.9 ± 16.6 HV, and 620.8 ± 57.1 HV, respectively. The microhardness of the GAL formed a three-level gradient in the range of 100 HV. The hardness of the surface strengthening layer was 85.1% higher than that of the substrate layer. The microhardness of each deposition layer fluctuated within a small range, which was due to the cyclic thermal process. The bead-overlapping area and the interlayer area underwent secondary remelting, resulting in grain growth and hardness reduction in the local area. The hardness at the interface of A, B, and C was between the hardness of the materials on both sides and smoothly increased.

**Figure 11:**
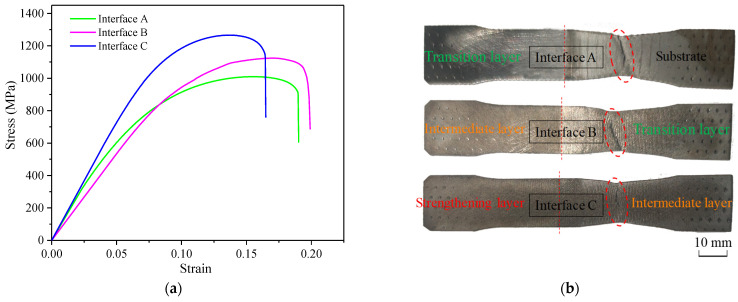
Bonding strength of gradient interfaces. (**a**) Stress–strain curves of the tensile specimens. (**b**) Image of fracture specimens after tensile test. The red circle outlines the location of the fracture.

The bonding strength of the gradient interfaces was tested, and the results are shown in Figure 11 and Table 5. The ultimate strength values of the substrate–transition layer, transition–intermediate layer, and intermediate–strengthening layer interfaces were 1007 MPa, 1123 MPa, and 1265 MPa, respectively. The yield strength values of the substrate–transition layer, transition–intermediate layer, and intermediate–strengthening layer interfaces were 672 MPa, 809 MPa, and 916 MPa, respectively. As far as can be judged from Figure 11b, the gradient interfaces remained intact, while the tensile fractures in these three specimens were all located rather far from the interface. In other words, the adjacent layers were firmly bonded at the interface, and the interfacial bonding strength was higher than the material in the adjacent layer that has lower strength. Additionally, the tensile elongation values of the substrate–transition layer, transition–intermediate layer, and intermediate–strengthening layer interfaces were 18.8%, 19.5%, and 16.2%, respectively. Compared with the substrate metal, the additive material of the transition layer showed a simultaneous increase in both ultimate strength and tensile elongation by 10.3% and 3.6%. On the one hand, the increased content of Cr and Mo in the transition layer acted as a solid solution strengthening, and a few fine, tempered martensite structures distributed in the transition layer also had a certain high strength. On the other hand, the increased content of Ni in the transition layer hindered the nucleation and growth of carbides and refined their size, thereby improving the elongation of the transition layer.

The microstructure of the GAL steel exhibited a distinct variation pattern from the bottom to the top layer, which was influenced by an increase in the alloying element content and an acceleration of the cooling rate. Specifically, the transition layer was predominantly composed of bainite, the intermediate layer exhibited a mixed structure of martensite and bainite, and the strengthening layer showed a fully martensitic structure. As a result, the microstructure of the GAL steel underwent a transformation process from bainite, which possessed good strength-toughness, to martensite, which had superior strength, with an increasing gradient from the bottom to the top layer. The mechanical properties of the GAL steel showed a corresponding gradient increase in both hardness and strength, which met the performance requirements of the working surface of hot forging dies.

### 3.3. Impact Property of Gradient Interfaces

**Figure 12:**
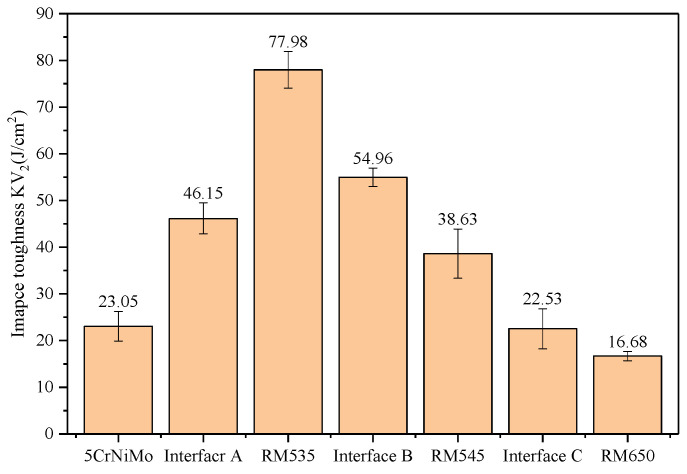
Impact toughness of gradient interfaces and deposition layers.

The impact energy per unit area of the deposition layers and the interfaces is illustrated in Figure 12. The impact toughness values of the substrate layer, transition layer, intermediate layer, and strengthening layer were 23.05 J/cm^2^, 77.98 J/cm^2^, 38.63 J/cm^2^, and 16.68 J/cm^2^, respectively. The impact values toughness of interfaces A, B, and C were 46.15 J/cm^2^, 54.96 J/cm^2^, and 22.53 J/cm^2^, respectively. It is clear that the impact toughness of the GAL showed a decreasing gradient trend and that the impact toughness of the transition layer was significantly higher than that of the substrate layer. As previously noted in the analysis of microhardness (Figure 10) and strength (Table 5), which indicated that the strength and hardness of the transition layer were better than those of the substrate layer, the toughness was also greatly improved. During the service of hot forging dies, the surface and interior of the die were subjected to different stresses and strains due to the uniform mechanical properties of a single material. This may result in an uneven distribution of stress and, ultimately, lead to failure of the die. Gradient materials had high surface hardness and good internal toughness. This material structure could reduce the unevenness of working stress between the surface and interior of the die. This was because the high surface hardness of the die could withstand higher stress, while the good internal toughness could better absorb and distribute stress. Therefore, the use of gradient materials for die repair could result in a more uniform stress distribution, reducing stress unevenness and improving the service life of dies.

**Figure 13:**
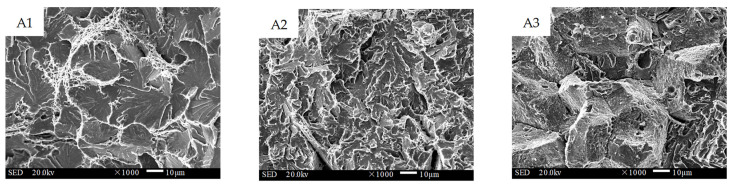
Impact fracture morphology of interface A between the substrate and the transition layer (1000×).

**Figure 14:**
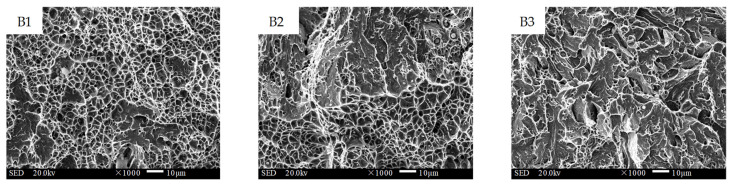
Impact fracture morphology of interface B between the transition and the intermediate layer (1000×).

**Figure 15:**
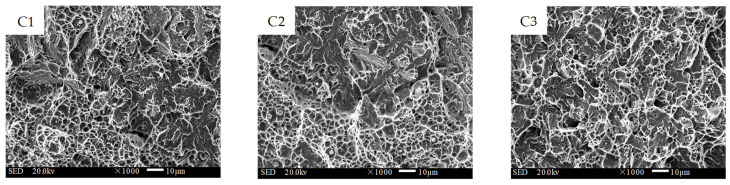
Impact fracture morphology of interface C between the intermediate and the strengthening layer (1000×).

Figure 13, Figure 14 and Figure 15 present the fracture morphology of interfaces A, B, and C, respectively. A1 was located near the substrate layer at interface A, and the micro-morphology was mainly a fan-shaped cleavage section with a small number of tearing edges and micro-hole dimples. The central area, A2, of interface A showed a quasi-cleavage fracture with no obvious boundary line, indicating that the bonding area was well fused and that the change gradient of the element composition and properties between the substrate layer and the transition layer was small. On the other hand, A3, the part of interface A close to the transition layer side, was characterized by huge blocky bulges with a few shallow and small dimple patterns on the crystal plane, indicating an intergranular mixed fracture. The abundant diffusion of C in the substrate material significantly reduced the toughness of the interfacial fusion zone in this area. Area B1, located close to the transition layer side, displayed an equiaxed orthogonal dimple with a large area of distribution. The microstructural analysis revealed that the grains in this area were dense, with fewer precipitates at the grain boundary, resulting in excellent toughness. The central area, B2, exhibited an obvious boundary line, where the lower part was a ductile fracture and the upper part was a quasi-cleavage fracture. In comparison to interface A, the differences in the type and content of elements of the two materials at interface B were fewer, and the boundary line of the interfacial fusion zone was more distinct. The boundary line could also be observed at interface C (shown in Figure 15C2). Although both C1 and C3 exhibited a quasi-cleavage fracture morphology, C1 had more dimples and better toughness. In contrast, C3 was dominated by cleavage facets, with unevenly distributed microporous dimples around the cleavage plane.

## 4. Production Application

**Figure 16:**
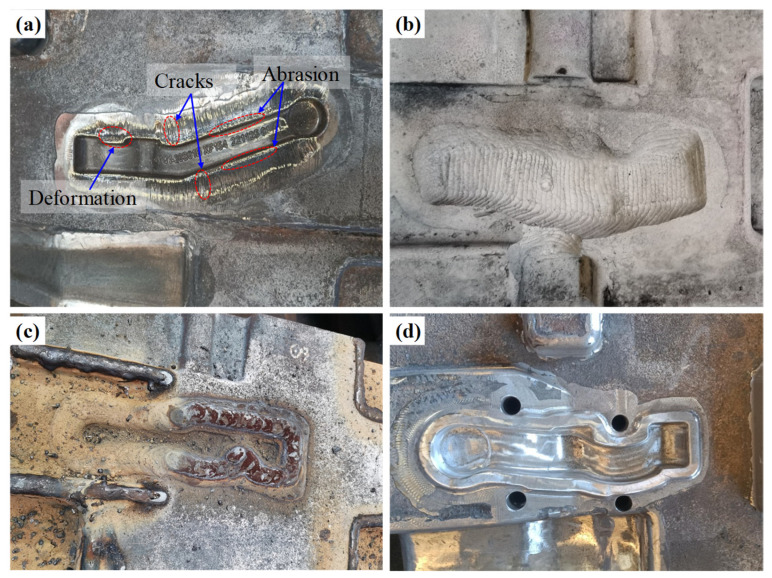
Production application of hot forging die remanufacturing: (**a**) the failed hot forging die; (**b**) removal of failure zone using carbon arc gouging; (**c**) wire and arc additive remanufacturing with multiple materials; (**d**) post-machining and polishing.

**Figure 17:**
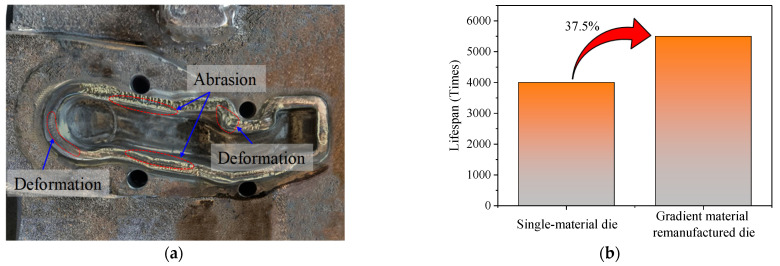
Lifespan comparison between the single-material die and gradient material remanufactured die. (**a**) The failed hot forging die using gradient material remanufacturing. (**b**) The improvement of service lifespan.

To evaluate the feasibility of manufacturing and the overall performance of the gradient layers of the die, a hot forging die for a vehicle control arm was remanufactured in a forging enterprise, as depicted in Figure 16. As seen in Figure 16a, the surface of the die exhibited numerous cracks and large deformations, and the edges of the cavity were severely abraded. This forging die had to be repaired to prolong its service life. Therefore, gradient wire arc additive remanufacturing was conducted for this hot forging die. First, the failure zone was removed (Figure 16b) using carbon arc gouging so that the designed remanufacturing zone could be obtained. Then, the remanufacturing zone of the hot forging die was divided into three deposition layers (as illustrated in Figure 3). Subsequently, wire arc additive remanufacturing with multiple materials (transition, intermediate, and strengthening layers) was performed on the die cavity, as revealed in Figure 16c. Finally, post-machining and polishing were carried out to obtain the required die cavity, as displayed in Figure 16d. In practical production applications, Figure 17a presented the failed hot forging die using gradient material remanufacturing after a period of service. The original single-material die had an average lifespan of 4000 pieces, while the die remanufactured using the proposed method had an average lifespan of 5500 pieces. There were several deformations on the surface of the die and serious abrasion on the edge of the cavity after serving 5500 pieces. Compared with the failed single-material die (Figure 16a), the failed die remanufactured with gradient materials was characterized by minimal cracks on its surface (Figure 17a) and an increased lifespan of 37.5%, demonstrating the superiority of the multi-material gradient additive remanufacturing approach.

## 5. Conclusions

A gradient multi-material wire arc additive remanufacturing method for hot forging dies was proposed in this paper. The remanufacturing zone of a hot forging die was divided into three deposition layers based on temperature and stress analyses. Experiments on a 5CrNiMo substrate were conducted using wire arc additive manufacturing with gradient materials. The microstructure, microhardness, bonding strength, and impact property of gradient interfaces were evaluated and discussed. The conclusions can be summarized as follows.

(1) Gradient interfaces A, B, and C were bonded without defects such as cracks, slag inclusions, or voids. The transition layer was predominantly composed of bainite, the intermediate layer exhibited a mixed structure of martensite and bainite, and the strengthening layer showed a fully martensitic structure. The microstructure of the gradient additive layers presented a gradient transformation process from bainite to martensite. Stable metallurgical bonding between the GAL and the substrate layer formed due to the sufficient interdiffusion of alloying elements during depositing.
(2) The microhardness gradually increased from the substrate layer to the surface strengthening layer, forming a three-level gradient in the range of 100 HV. The strength of the substrate–transition layer, transition–intermediate layer, and intermediate–strengthening layer interfaces exhibited a reasonable increase, and the additive material of the transition layer showed an increase in both strength and tensile elongation at the same time. The fracture position of the tensile specimens was located rather far from the interface, which indicated that the gradient interfaces obtained high-strength metallurgical bonding. The impact toughness values of interfaces A, B, and C were 46.15 J/cm^2^, 54.96 J/cm^2^, and 22.53 J/cm^2^, and the impact fracture morphology ranged from ductile fracture to quasi-cleavage fracture.
(3) In practical production applications, the hot forging dies using the gradient multi-material wire arc additive remanufacturing method had an average lifespan of 5500 pieces, an increase of 37.5% compared with the original single-material die, which indicated the superiority of the proposed multi-material gradient additive remanufacturing approach.

## Figures and Tables

**Table 1:** Parameters of forging process in the simulation.

Parameter	Value
Ambient temperature (°C)	25
Initial forging temperature of workpiece (°C)	1150
Pre-heating temperature of die (°C)	200
Forming speed of press machine (mm/s)	60
Heat convection coefficient (N/s/mm/°C)	0.02
Radiation coefficient	0.3
Friction coefficient	0.3

**Table 2:** Time and heat conduction coefficient of each stage in the forging process.

Parameter	Time (s)	Heat Conduction Coefficient (N/s/mm/°C) [16]
Workpiece placing	2	1
Forging	0.5	11
Pressure maintenance	1	2
Cooling and lubrication	2	8

**Table 3:** Chemical composition (wt.%) of 5CrNiMo steel and additive layers.

Additive Layer	Material	Element												
		C	Si	Mn	Cr	Mo	Ni	Ti	V	W	Ta	P	S	Fe
Substrate	5CrNiMo	0.55	0.32	0.62	0.76	0.23	1.61	~	0.02	~	~	0.01	0.01	Bal.
Transition layer	RM535	0.06	0.32	1.20	1.71	1.65	2.60	0.04	0.01	0.04	0.03	0.01	0.01	Bal.
Middle layer	RM545	0.09	0.45	1.29	8.68	2.26	1.98	0.06	0.02	0.04	0.18	0.01	0.01	Bal.
Strengthening layer	RM650	0.18	0.44	1.36	10.96	3.10	1.92	0.06	0.03	0.04	0.22	0.01	0.01	Bal.

**Table 4:** Welding process parameters.

Material	Arc Voltage (V)	Arc Current (A)	Wire Feed Speed (m/min)	Welding Speed (mm/s)	Single Bead Width (mm)	Single Bead Height (mm)
RM535	21.5	233.0	3.7	7.0	6.5	2.7
RM545	21.5	233.0	3.7	7.5	6.8	2.5
RM650	21.5	233.0	3.7	7.0	6.7	2.7

**Table 5:** Tensile properties of gradient interfaces.

Interface	Ultimate Strength (MPa)	Yield Strength (0.2% Strain Offset [35], MPa)	Elongation (%)	Tensile Fracture Position
Interface A	1007 ± 9	672 ± 18	18.8 ± 1.0	Substrate layer
Interface B	1123 ± 23	809 ± 15	19.5 ± 0.3	Transition layer
Interface C	1265 ± 26	916 ± 12	16.2 ± 0.8	Intermediate layer

## Data Availability

Not applicable.
